# A Mixing Model for Describing Electrical Conductivity of a Woven Structure

**DOI:** 10.3390/ma15072512

**Published:** 2022-03-29

**Authors:** Magdalena Tokarska

**Affiliations:** Faculty of Material Technologies and Textile Design, Institute of Architecture of Textiles, Lodz University of Technology, 90-924 Lodz, Poland; magdalena.tokarska@p.lodz.pl

**Keywords:** Archie’s law, mixing model, electrical conductivity, woven structure, composite

## Abstract

The main aim of the research was to describe electro-conductive woven structures by specifying the phases’ exponents using the generalised Archie’s law. Special woven structures were designed to transfer Archie’s model to the textile object. The woven structure was treated as a complex multiphase mixture. The structure was composed of two conducting phases (strips and strip contacts) and one non-conducting phase (pore space). It was found that the designed structures were characterised by the phases’ exponents that exceeded the value of 2, which denoted low connectivity in the conductive phases. A qualitative and quantitative description of the woven structure was feasible, i.e., the connectedness and the connectivity, respectively. The connectedness of both of the phases was dependent on the material from which the structure was designed. The fraction of each of the phases involved in the current conductivity was important. The connectivity connected with structure density, in varying degrees, affected the electro-conductive properties of the woven structure. It was important how the phases were arranged in the whole composite. It was found that the strips’ contact phases played an important role in the structure of the composite.

## 1. Introduction

Due to their electrical properties, woven and knitted fabrics belonging to flat textile objects can be used as textile sensors [1,2,3,4]. Most textile-based sensors rely on a change in electrical resistance. To predict their conductivity, models based on equivalent resistance schemes are used [5,6,7,8]. The fabric is seen from an electrical point of view as an electrical circuit composed of connected resistors and a battery. Yarns are ideal resistors of known resistance. The simulated structure was regular so the resistive model was a simplified model of the textile object, which was much more complex and showed the anisotropy of the electrical properties [9,10,11,12]. The electrical conductivity of flat textile materials results from the electrical conductivity of their components, i.e., fibres and yarns and contact resistances resulting from interlaced yarns [8,13,14,15]. Woven fabric can be compared to metal-dielectric composites where conductive linear components (yarns) create a system of empty spaces filled with dielectric air [10,14]. To predict the resistivity of fabrics, the McLachlan equation described in [16,17] was modified [14]. A prediction level in the range of 83–88% for fabrics with a surface percentage covered with yarns above 96% was obtained [14]. Contact resistance resulting from the interlaced yarns was not taken into consideration.

Many types of mixing models are used to determine the electrical properties of porous materials. One of them is Archie’s law [18] extended for n phases and known as the generalised Archie’s law [19]. Archie developed an empirical quantitative relationship between the porosity, electrical conductivity, and brine saturation of rocks. The law describes the relationship of the conductivity of a clean reservoir rock to its porosity and the conductivity of phase (e.g., fluid) that completely saturates the pore space, and is given as follows [19]:

*σ* = *σ_f_ ϕ^m^* (1)

where *σ* is the bulk effective conductivity of the rock, *σ_f_* is the conductivity of the fluid occupying the pores, *ϕ* is the volume fraction of the fluid phase, and *m* is the cementation exponent. Due to the fluid occupying the pores, and the pore space being fully saturated, *ϕ* is identical to the porosity.

The parameter *G* = *σ*/*σ_f_* = *ϕ^m^*, called the connectedness of the porous medium, was defined by Glover and Walker [20]. The connectedness of a given phase is a measure of the availability of pathways for conduction through that phase. The connectivity defined as *χ* = *ϕ^m^*^−1^ is a measure of how the pore space is arranged [19]. A small exponent *m* (below 2) occurs for high connectivity phases [21]. Pores that are well connected provide an efficient pathway for the medium flow. A large exponent *m* (equal to or above 2) occurs for low connectivity phases. It was noticed that a cementation exponent *m* depends on the shape and type of the sedimentary rock grains, the shape and type of pores, specific surface area, tortuosity, anisotropy, and compaction [22]. Archie’s law was used to predict the conductivity-brine volume trend for sandstone [23], and to characterise sedimentary rock formations, i.e., carbonate rocks, which are prone to develop a wide variety of pore structures [22]. The law modifications were presented for tight and clay-rich reservoirs to describe electrical conductivity in such reservoirs [24]. The generalised Archie’s law was also used for the modelling of electro-conductive properties of woven structures [25]. Parameters such as connectedness and connectivity, determined for conducting phases, enabled an explanation of the phenomenon of current conduction in a woven structure. It was found that a decrease in the connectedness of strips and strip contacts’ phases could be obtained by adding another component to the woven structure, which would reduce the conductivity of the whole structure. The larger values of connectivity for the strips’ phase compared to the connectivity of the strip contacts’ phase meant that the strips’ phase (in terms of their quantity) had a greater effect on the conductivity of the woven structure than the strip contacts’ phase.

Archie’s law is not applicable if there are two or more conducting phases, or if the conducting phase does not fill the pore space. Due to the occurrence of porous media consisting of solid and fluid phases of known conductivities, volume fractions, and distributions, Archie’s law was modified by Glover et al. for two [21], and *n* conducting phases [19]. The generalised Archie’s law is as follows:

(2) $$\sigma =\overset{n}{\underset{i=1}{\sum }}\sigma_{i}\phi {}_{i}^{m_{i}}$$

where *σ* is the mixing model conductivity, *σ_i_* is the *i*-th phase conductivity, *ϕ_i_* is the *i*-th phase volume fraction, *m_i_* is the *i*-th phase exponent, and *n* is the number of conducting phases.

It was stated that the sum of the volume fractions of all of the phases in a porous medium (a rock) must equal unity. Each of the *n* phases that occupy the rock must share the same total space. This means that an increase in the connectedness of one of the phases must lead to a reduction in the connectedness of at least one of the other phases [19].

In the generalised Archie’s law, phase exponent *m* < 1 represents a phase with a high degree of connectivity [19,26]. A value of *m* ≈ 1 can be observed for rocks with a low porosity but a well-developed fracture network, the network then has fairly direct flow paths. A phase exponent *m* ≈ 2 means that the phase is partially connected in a similar way as in sandstone [23,24,27]. A higher value of *m* represents lower phase connectivity as in the case of vuggy limestone [22]. The classical and generalised Archie’s laws share the property that the exponents modify the volume fraction of the relevant phase concerning the total volume of the rock.

The main aim of this research was to describe electro-conductive woven structures by specifying the phases’ exponents using the generalised Archie’s law. This meant that by designing the same structure, but from other components, you could predict the conductivity of the new structure based on parameters determined from the mixing model. The law can be applied to objects containing pores filled with phases. Therefore, special woven structures were designed to transfer Archie’s model to the textile object. New structures were composed of interlaced strips cut from fabrics. Such a structure was treated as a complex multiphase mixture containing two phases and a matrix. The strips and the strip contacts were phases that allowed current to be conducted in the composite. The remaining part was pore space, corresponding to the non-conducting matrix. So, the pores of the woven structure were not identical with the pores of Archie’s model, but were opposite to the matrix. This comparison was an original and innovative approach to the woven structure for modelling its electro-conductive properties. The features of the fabric’s linear elements and the number of their contacts seemed to be important in controlling the electrical conductivity of the woven structure.

## 2. Materials

Three commercially available electro-conductive woven fabrics were chosen to construct new woven structures. The raw material composition of the fabrics is presented in Table 1. The fabric denoted as S1 was purchased from Laird™ (ABC Elektronik Sp.z o.o., Gorlice, Poland), and the fabrics denoted as S2 and S3 were purchased from Soliani™ (Como, Italy). According to datasheets, the surface resistivity of S1 was below 0.07 Ω/sq, and for S2 and S3 did not exceed 0.40 Ω/sq.

Parameters of the textile materials (woven fabrics) are presented in Table 2.

The mean values of quantities such as thickness, areal density, and bulk density, and their relative expanded uncertainties *U* (given in parentheses in Table 2) for confidence level equal to 0.95, were calculated according to the following equation [28]:

*U* = *k_p_ u_c_*(*y*) (3)

wherein

(4) $$u_{C}^{2}\left(y\right)=\overset{N}{\underset{i=1}{\sum }}\left[{\left(\frac{\partial f}{\partial x_{i}}\right)}^{2}\left(u_{A}{}^{2}\left(x_{i}\right)+u_{B}{}^{2}\left(x_{i}\right)\right)\right]$$

where *k_p_* is the coverage factor (for confidence level equal to 0.95, the coverage factor equals 2), *u_C_*(*y*) is the combined variance, *u_A_*(*x_i_*) is the Type A standard uncertainty estimated from independent repeated observations, *u_B_*(*x_i_*) is the Type B standard uncertainty evaluated by scientific judgment based on all of the available information on the possible variability of input quantity, *N* is the number of independent input quantities *x_i_* with *i* = 1, 2, …, *N*, *y* is the estimate of an output quantity, and *f* is the functional relationship between input and output quantities (for direct measurements).

Measurements of thickness and mass were repeated five times. Measurements of yarn densities were repeated three times. A rectangular distribution of possible values for the calculation of the Type B uncertainty was assumed [29].

**Figure 1:**
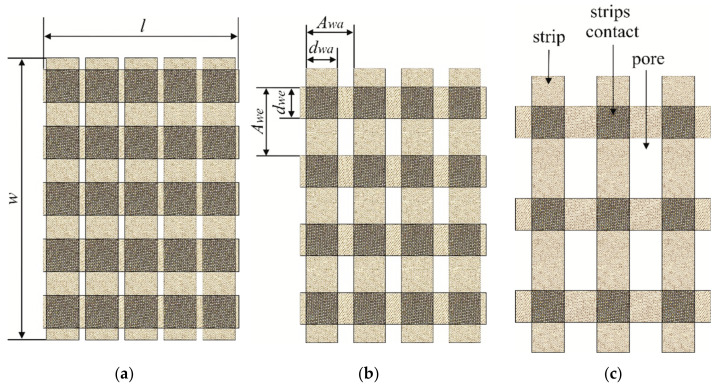
Woven structure types designed from strips: (**a**) 5 × 5; (**b**) 4 × 4; (**c**) 3 × 3.

Strips of length 15 cm and two different widths (1.0 cm and 1.5 cm) were cut from fabrics S1, S2, and S3. New plain weave structures composed of the same strips and the same fabric were designed for research purposes. Another woven structure was handmade from interlaced strips based on a specific report. The three structures for strip width equal to 1.5 cm are shown in Figure 1. The fabric components are presented in Figure 1c. Three more structures differing in the width of the strips (1.0 cm) were also designed. The number *k* of strips in the weft and warp directions was the same for the chosen structure but all the structures differed in strip density (strips per unit of length). The structure dimension *l* × *w* was assumed for the target research, where *l* was the structure length (wherein *l* = 9 cm), and *w* was the structure width (wherein *w* = 13 cm) (see Figure 1a).

The designed woven structures were characterised by parameters such as the spacing of warp and weft strips *A_wa_* and *A_we_*, respectively, and the width of warp and weft strips *d_wa_* and *d_we_*, respectively [10,30]. The parameters were determined and are shown in Figure 1b for the second structure. Parameters of six designed woven structures are presented in Table 3.

Based on parameters of the designed woven structures (Table 3), fractions of components (i.e., strips, strips contacts, and pores) in the whole woven structure and percentage surface cover were determined and are presented in Table 4. The fraction of the component was calculated as a quotient of the particular component area and the whole area of the woven structure (*l* × *w*), wherein *C_th_* + *C_cont_* + *C_p_* = 1. The fraction of strips applied only to strips, excluding their contact surfaces.

Eighteen woven structures were prepared using three different woven fabrics S1, S2, and S3.

## 3. Methods

The generalised Archie’s law (see Equation (2)) for the woven structure can be rewritten as:

(5) $$\sigma_{str}=\sigma_{th}\phi {}_{th}^{m_{th}}+\sigma_{cont}\phi {}_{cont}^{m_{cont}}$$

where *σ_str_* is the conductivity of the woven structure, *σ_th_* is the conductivity of the strips phase, *σ_cont_* is the conductivity of strip contacts’ phase, *ϕ_th_* is the area fraction of the strips’ phase, *ϕ_cont_* is the area fraction of the strip contacts’ phase, *m_th_* is the strips’ phase exponent, and *m_cont_* is the strip contacts’ phase exponent. Equation (5) is valid for *σ_th_*, *σ_cont_*, *m_th_*, *m_cont_* > 0, and *ϕ_th_*, *ϕ_cont_* ∈ (0,1), wherein *ϕ_th_* + *ϕ_cont_* = 1. Due to the thickness of strips being very small compared to the dimensions of strips and contact strips, instead of the volume fraction of each phase, the area fraction was taken into consideration in Equation (5). Based on the assumption that each conducting phase was fully saturated, i.e., identical to the area fraction of the phase, and detailed analysis for two phases considered by Glover [19], the following equality holds:

(6) $$\left(-\frac{\phi {}_{th}^{2}}{2}\right)m_{cont}^{2}+\left(\phi {}_{th}-\frac{\phi {}_{th}^{2}}{2}\right)m_{cont}-\phi_{th}^{m_{th}}=0$$

If the conductivities of the individual phases and their fractions are known, the application of simultaneous Equations (5) and (6) enables the determination of the strips’ phase exponent and the strip contacts’ phase exponent. As it was stated that the sum of the area fractions of all of the phases in the composite must equal unity, the same assumption was adopted for the sum of the connectedness of all of the phases.

To describe the woven structure, connectedness and connectivity were adopted. The connectedness of the strips’ phase is given by

(7) $$G_{th}=\frac{\sigma {}_{str}}{\sigma {}_{th}}$$

While the connectivity is given by

(8) $$\chi {}_{th}=\phi_{th}^{m_{th}-1}$$

The connectedness of the strip contacts’ phase is given by

(9) $$G_{cont}=\frac{\sigma {}_{str}}{\sigma {}_{cont}}$$

While the connectivity is given by

(10) $$\chi {}_{cont}=\phi_{cont}^{m_{cont}-1}$$

**Figure 2:**
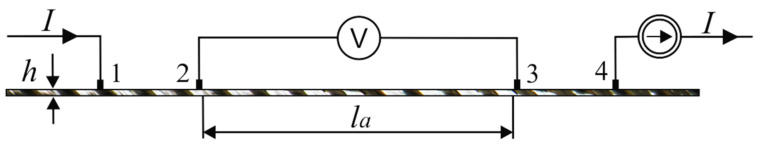
Strip resistance measurements (the side view).

The resistance of the strips was determined based on the four-electrode method [31]. Parallel brass plates were used as electrodes. Current I was injected through the two outer electrodes (1 and 4) and voltage drop UI between the two inner electrodes (2 and 3) was measured (Figure 2). The resistance could then be calculated.

Conductivity *σ_th_* of the strip could be determined using the following equation:

(11) $$\sigma_{th}=\frac{l_{a}}{dhR}$$

where *R* is the strip resistance, *l_a_* is the voltage electrodes spacing (*l_a_* = 5 cm), *d* is the strip width (*d* = 1.0 cm or *d* = 1.5 cm), and *h* is the strip thickness (corresponding to the fabric thickness).

Measurements were conducted for 10 strips cut from the same woven fabric. All measurements were repeated three times.

**Figure 3:**
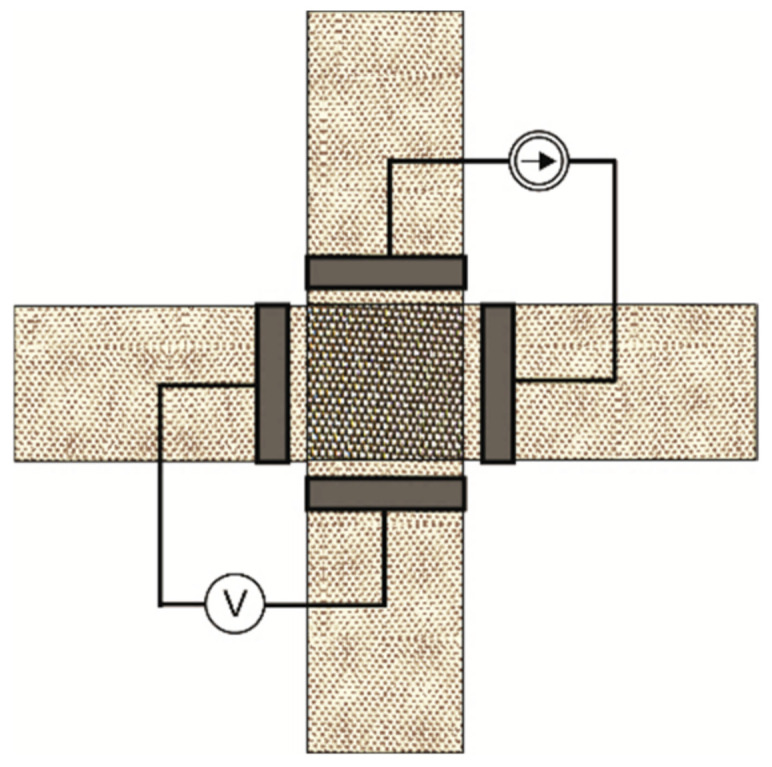
Strip contact resistance measurements (the top view).

The resistance of strip contacts was determined using the four-electrode method described in detail in [13,32]. Brass plates were used as electrodes. The idea of the measurement method is presented in Figure 3. An initial load of 5 cN was applied to avoid the strips moving relative to each other. Based on the indirect method, resistance could be determined using Ohm’s law.

As shown in Figure 3, the contact surface was in the shape of a square with each side being 1.0 cm or 1.5 cm depending on the width of the strips. Measurements were conducted for three pairs of strips cut from the same woven fabric. All measurements were repeated three times.

Conductivity *σ_cont_* of the strip contact could be determined using the following equation:

(12) $$\sigma_{cont}=\frac{1}{2Rh}$$

where *R* is the resistance of the strips contact, and *h* is the strip thickness (double fabric thickness was assumed in Equation (12)).

**Figure 4:**
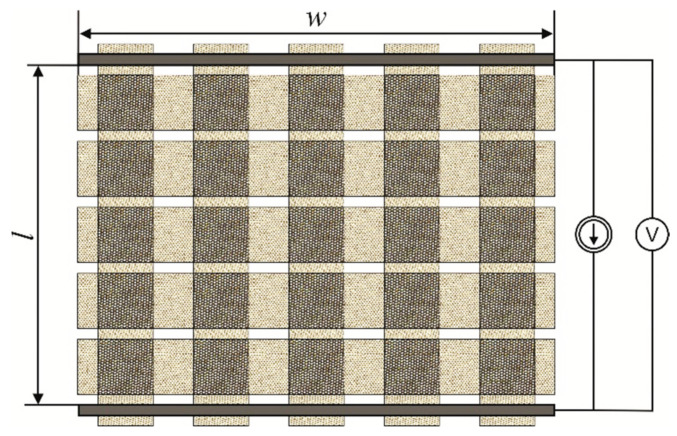
Woven structure resistance measurements (the top view).

Resistance measurements of the designed woven structure were performed by the four-wire method using two electrodes [33]. Brass plates were used as electrodes. The direct measurement method is presented in Figure 4. Measurements were carried out in the weft direction.

It was assumed, that the yarn deformations in the strips forming the woven structure had a negligible effect on the resistance measurements of the structure. Conductivity *σ_str_* of the woven structure could be determined using the following equation:

(13) $$\sigma_{str}=\frac{l}{wh^{\prime }R}$$

wherein

*h’* = 2 *hC_cont_* + 1 *hC_th_* + 0 *hC_p_* = 2 *hC_cont_* + *hC_th_* (14)

where *R* is the woven structure resistance, *l* is the spacing of the electrodes (*l* = 9 cm), *w* is the structure width (*w* = 13 cm), *h’* is the resultant fabric thickness, *C_cont_* is the fraction of strip contacts in the whole woven structure, *C_th_* is the fraction of strips in the structure, and *C_p_* is the fraction of pores in the structure, called the porosity.

A DC power supply Agilent E3644A (Agilent, Santa Clara, CA, USA) was used as an ammeter. The resolution of the ammeter was 0.001 A. A multimeter Agilent 34410A (Agilent) was used as a voltmeter. The resolution of the voltmeter was 0.0001 V.

## 4. Results and Discussion

Measurements of resistance of strips and strip contacts were carried out in standard atmospheric conditions according to the standard [34]. Conductivities were calculated according to Equations (11)–(14). Received results are given in Table 5. The coefficient of variation determined for conductivities is given in parentheses. Area fractions of phases *ϕ_th_* and *ϕ_cont_* were also determined and are given in Table 5.

Solving the simultaneous Equations (5) and (6) in Mathematica^®^ 8, the exponents *m_th_* and *m_cont_* of phases were determined. Based on Equations (7)–(10) the connectedness and connectivity for phases of the woven structures were determined. The results are juxtaposed in Table 6.

First of all, linear regression analysis using Statistica^®^ 13 was performed assuming a significance level *α* = 0.10 and Pearson’s correlation coefficient *R_P_* was calculated (Table 7). A significance level equal to 0.10 meant that we were willing to make 10 mistakes out of 100 tests. In this situation, it was easier to reject the null hypothesis. This approach made it possible to detect potential relationships and analyze them, and determine whether they made sense from the point of view of designing the woven structure and its electro-conductive properties.

The initial analysis indicated relationships^(1)^ between the conductivity of the whole structure *σ_str_* and conductivities of phases *σ_th_* and *σ_cont_*, wherein the relationships *σ_str_* and *σ_th_* were stronger (*R_P_* = 0.998), while *σ_str_* and *σ_th_* were weaker (*R_P_* = 0.619), assuming a significance level *α* = 0.10. No significant dependences were observed between the parameter *C_str_* connected with the structure and parameters *G_th_* and *G_cont_* connected with electro-conductive features of the used materials.

There were significant correlations^(2)^ between the parameters *ϕ_th_*, *m_th_*, *χ_th_*, and *ϕ_cont_*, *m_cont_*, *χ_cont_* resulted from the fact that they met Equation (10). The parameters were directly related^(3)^ to the woven structure; no relation to the conductivities of phases was observed.

Next, statistical analysis was performed using Statistica^®^ 13 based on the Kruskal–Wallis (K–W) test [35]. The determined *p*-value was compared with the critical value *α* for rejecting the null hypothesis. If *α* was less than the *p*-value, the null hypothesis was not rejected. When the K–W test led to significant results, at the assumed significance level *α*, at least one group was different from the other groups. To identify the particular differences between pairs of groups, a post hoc test was used.

The nonparametric statistical procedure was used for comparing chosen parameters (*G_th_* and *G_cont_*) in three independent groups, i.e., woven structures designed from the same electro-conductive woven fabric (S1, S2, or S3). The test was performed assuming *α* = 0.10. Results of statistical analysis are presented in Table 8.

**Figure 5:**
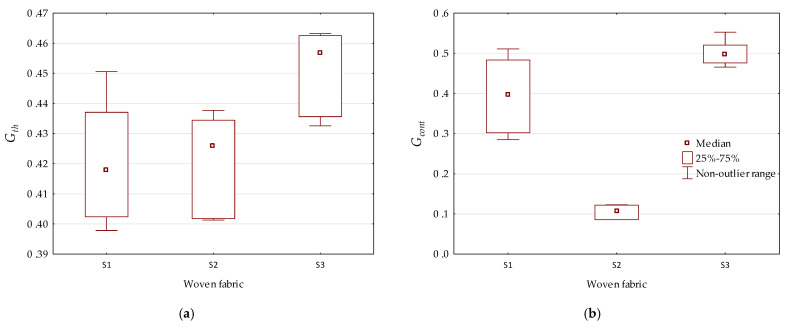
Influence of material on phase connectedness: (**a**) strips’ phase; (**b**) strip contacts’ phase.

**Figure 6:**
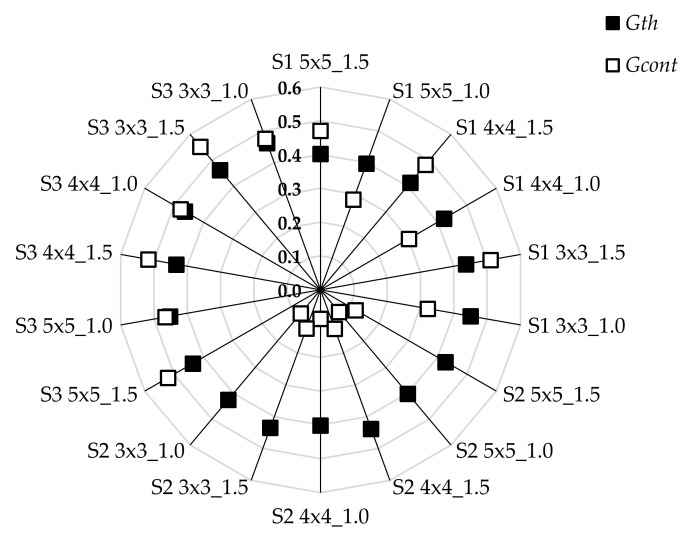
Connectedness of strips’ and strip contacts’ phases.

It was found that there were significant differences in groups for both the *G_th_* and *G_cont_* parameters. This meant that the connectedness of both of the phases was dependent on the material (electro-conductive woven fabric) from which the structure wa s designed (see Figure 5). It was noted that the range of connectedness change *G_th_* was not large compared to *G_cont_*. This was due to the design assumptions of woven structures. A greater variation (59%) of the area fraction of the strip contacts’ phase *ϕ_cont_* compared to the variation (8%) of the area fraction of the strips’ phase *ϕ_th_* was observed (Table 5). Therefore, each phase fraction was important in the conductivity of the current through the woven structure. The connectedness could be considered in terms of woven structure quality. Electrical conduction of a woven structure depends on the phases, which are pathways enabling current conduction. Phases, in varying degrees, affect the electro-conductive properties of a woven structure as shown in Figure 6. It was noticed that the contribution of the strip contacts’ phase in the conductivity of woven structure for fabric S2 was lower than that in the case of fabrics S1 and S3. The surface of woven fabric S2 seemed to be much smoother than the surfaces of the remaining fabrics. The resistivity of strips cut from fabric S2 was higher than the resistivity of the strip contacts.

The nonparametric statistical procedure was used for comparing chosen parameters (*m_th_*, *m_cont_*, *χ_th_*, and *χ_cont_*) in three independent groups, i.e., woven structures designed with the same density of strips (5 × 5, 4 × 4 or 3 × 3). Results of statistical analysis conducted for significance level *α* = 0.10 are presented in Table 9.

It was found that there were significant differences in groups for three of the four parameters. The phases’ exponents *m_th_*, *m_cont_*, differed significantly in two groups of structures: 3 × 3 and 5 × 5. The relationship of the connectivity *χ_th_* with the designed three different structures was not found in contrast to *χ_cont_*. As mentioned earlier, this might be due to the low variation of the area fraction of the strips’ phase *ϕ_th_*.

**Figure 7:**
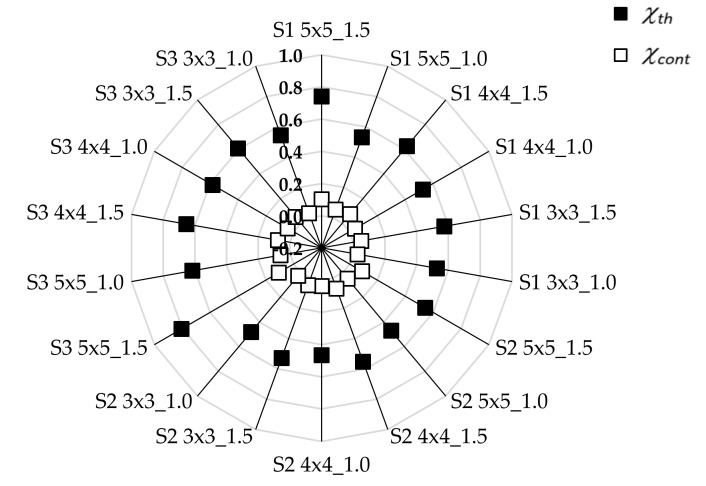
Connectivities of strips’ and strip contacts’ phases.

The connectivity could be considered in terms of woven structure quantity. Structure density, in varying degrees, affects the electro-conductive properties of a woven structure. It is important how phases are arranged in the whole composite. Connectedness for both phases that characterise woven structures is shown in Figure 7. Larger values of *χ_th_* compared to *χ_cont_* were observed for all woven structures. The results show that the strip contacts’ phase played an important role in the structure of the composite.

**Figure 8:**
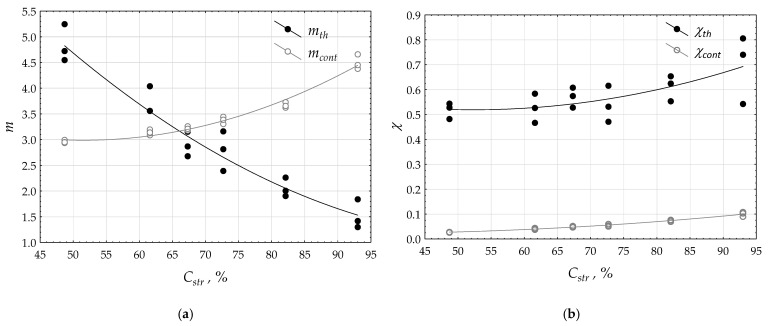
Influence of percentage surface cover on: (**a**) phase exponent; (**b**) phase connectivity.

Parameters *m_th_*, *m_cont_*, *χ_th_*, and *χ_cont_* were connected with the woven structure and especially with percentage surface cover *C_str_*, as shown in Table 7. Some dependences were observed and are presented in Figure 8.

The results of the measurements were approximated by quadratic *polynomials.* Coefficients of determination *R*^2^ were as follows for *m_th_* = *f(C_str_)* – *R^2^* = 0.908, and for *m_cont_* = *f(C_str_)* – *R^2^* = 0.854 (Figure 8a); for χ*_th_* = *f(C_str_)* – *R^2^* = 0.417, and for χ*_cont_ = f(C_str_)* – *R^2^* = 0.936 (Figure 8b). All the coefficients were significant at the 0.10 significance level. It was found that the percentage surface cover increase caused an increase in connectivity of each phase. An increase in percentage surface cover caused a decrease in the strips’ phase exponent and an increase in the strip contacts’ phase exponent. Each of the phases that occupy the composite must share the same total space. This meant that the increase in the fraction of one of the phases must lead to a reduction in the fraction of the second phase.

The mean values of phases’ exponents are presented in Table 10. Types of designed woven structures and the width of strips were taken into account. Variation coefficients are given in parentheses.

A wider range of phase exponent values was observed for the strips’ phase than for the strip contacts’ phase. The vast majority of phases’ exponents exceeded 2. It could therefore be concluded that the low connectivity of conductive phases in the composites occurred. The exception was the strips’ phase in the woven structure of which the percentage surface cover was *C_str_* = 93%. The percentage surface cover for the remaining woven structures was in the range of 49–82%. However, further research is needed in this area.

## 5. Conclusions

The generalised Archie’s law can be used for describing the electrical conductivity of a woven structure. Every phase occurring in a composite has a well-defined exponent. In general, it could be concluded that the designed structures were characterised by the phases’ exponents exceeding 2, denoting low connectivity of the conductive phases. A qualitative and quantitative description of the woven structure was feasible. The connectedness could be considered in terms of woven structure quality. The connectedness of both of the phases was dependent on the material from which the structure was designed. Electrical conduction of woven structures depends on the phases, which are pathways enabling current conduction. The fraction of each of the phases involved in the current conductivity is also important. The fraction of phase is connected with its connectivity. The connectivity can be considered in terms of woven structure quantity. Structure density, in varying degrees, affects the electro-conductive properties of a woven structure. It is important how phases are arranged in the whole composite. This meant that the strip contact’s phase plays an important role in the structure of the composite. By designing the same structure but from other components, you could predict the conductivity of the new structure based on parameters determined from the mixing model.

## Figures and Tables

**Table 1:** The raw material composition of woven fabrics.

Woven Fabric	S1	S2	S3
Raw material composition	100% polyamide woven fabric; nickel and copper metalised	100% polyester woven fabric; nickel metalised	100% polyester woven fabric; nickel metalised
Weave	Plain	Plain	Twill
Microscopic image with total visual magnification 30× ↓ the warp direction → the weft direction	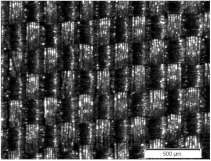	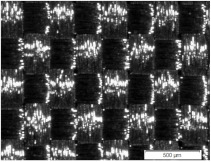	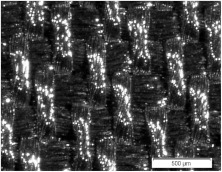

**Table 2:** Parameters of woven fabrics.

Woven Fabric	Thickness (mm)	Areal Density (g/m^2^)	Bulk Density (kg/m^3^)	Warp Density (Yarns/1 cm)	Weft Density (Yarns/1 cm)
S1	0.124 (10.5%)	86 (2.3%)	694 (10.8%)	57.0 (1.7%)	41.0 (1.2%)
S2	0.078 (15.4%)	75 (2.7%)	966 (15.8%)	40.0 (1.2%)	30.0 (1.7%)
S3	0.270 (5.6%)	152 (2.6%)	564 (5.8%)	47.5 (1.9%)	34.0 (1.7%)

**Table 3:** Parameters of designed structures.

*d_wa_* = *d_we_*	*k* × *k*	*A_wa_* (cm)	Pore Width ^1^ (cm)	*A_we_* (cm)	Pore Length ^2^ (cm)
1.5 cm	5 × 5	1.80	0.30	2.60	1.10
	4 × 4	2.25	0.75	3.25	1.75
	3 × 3	3.00	1.50	4.33	2.83
1.0 cm	5 × 5	1.80	0.80	2.60	1.60
	4 × 4	2.25	1.25	3.25	2.25
	3 × 3	3.00	2.00	4.33	3.33

^1^ Pore width equals *A_wa_*–*d_wa_*. ^2^ Pore length equals *A_we_*–*d_we_*.

**Table 4:** The fraction share of components in the whole woven structure.

*d_wa_* = *d_we_*	*k* × *k*	Fraction of Strips *C_th_* (-)	Fraction of Strip Contacts *C_cont_* (-)	Fraction of Pores *C_p_* (-)	Percentage Surface Cover *C_str_* (%) ^1^
1.5 cm	5 × 5	0.449	0.481	0.070	93
	4 × 4	0.513	0.308	0.179	82
	3 × 3	0.500	0.173	0.327	67
1.0 cm	5 × 5	0.513	0.214	0.273	73
	4 × 4	0.479	0.137	0.384	62
	3 × 3	0.410	0.077	0.513	49

^1^ Values calculated according to the equation: $C_{str}=\frac{A_{we}d_{wa}+A_{wa}d_{we}-d_{wa}d_{we}}{A_{wa}A_{we}}100$ [10].

**Table 5:** Characterisation of woven structures and their components.

Group	Sample	*ϕ_th_* (-)	*σ_th_* (Ω cm)^−1^	*ϕ_cont_* (-)	*σ_cont_* (Ω cm)^−1^	*σ_str_* (Ω cm)^−1^
A1	S1 5 × 5_1.5	0.483	2909.3 (6%)	0.517	2489.0 (30%)	1170.7 (9%)
	S2 5 × 5_1.5	0.483	617.5 (16%)	0.517	2202.8 (24%)	263.6 (8%)
	S3 5 × 5_1.5	0.483	140.7 (6%)	0.517	138.2 (38%)	61.3 (4%)
A2	S1 5 × 5_1.0	0.706	2291.1 (4%)	0.294	3200.2 (17%)	911.4 (5%)
	S2 5 × 5_1.0	0.706	712.1 (6%)	0.294	3338.7 (13%)	286.1 (7%)
	S3 5 × 5_1.0	0.706	150.3 (9%)	0.294	145.8 (11%)	67.9 (4%)
B1	S1 4 × 4_1.5	0.625	2909.3 (6%)	0.375	2489.0 (30%)	1203.2 (3%)
	S2 4 × 4_1.5	0.625	617.5 (16%)	0.375	2202.8 (24%)	270.3 (2%)
	S3 4 × 4_1.5	0.625	140.7 (6%)	0.375	138.2 (38%)	60.9 (3%)
B2	S1 4 × 4_1.0	0.778	2291.1 (4%)	0.222	3200.2 (17%)	966.7 (4%)
	S2 4 × 4_1.0	0.778	712.1 (6%)	0.222	3338.7 (13%)	285.7 (7%)
	S3 4 × 4_1.0	0.778	150.3 (9%)	0.222	145.8 (11%)	69.6 (9%)
C1	S1 3 × 3_1.5	0.743	2909.3 (6%)	0.257	2489.0 (30%)	1271.6 (2%)
	S2 3 × 3_1.5	0.743	617.5 (16%)	0.257	2202.8 (24%)	268.3 (3%)
	S3 3 × 3_1.5	0.743	140.7 (6%)	0.257	138.2 (38%)	65.1 (1%)
C2	S1 3 × 3_1.0	0.842	2291.1 (4%)	0.158	3200.2 (17%)	1032.2 (7%)
	S2 3 × 3_1.0	0.842	712.1 (6%)	0.158	3338.7 (13%)	302.3 (2%)
	S3 3 × 3_1.0	0.842	150.3 (9%)	0.158	145.8 (11%)	69.4 (1%)

**Table 6:** Characterisation of phases of woven structures.

Group	Sample	*m_th_* (-)	*G_th_* (-)	*χ_th_* (-)	*m_cont_* (-)	*G_cont_* (-)	*χ_cont_* (-)
A1	S1 5 × 5_1.5	1.414	0.402	0.740	4.453	0.470	0.103
	S2 5 × 5_1.5	1.840	0.427	0.542	4.659	0.120	0.089
	S3 5 × 5_1.5	1.297	0.436	0.806	4.379	0.521	0.108
A2	S1 5 × 5_1.0	2.814	0.398	0.532	3.388	0.285	0.054
	S2 5 × 5_1.0	3.161	0.402	0.471	3.445	0.086	0.050
	S3 5 × 5_1.0	2.393	0.452	0.616	3.305	0.466	0.059
B1	S1 4 × 4_1.5	2.002	0.414	0.624	3.653	0.483	0.074
	S2 4 × 4_1.5	2.262	0.438	0.553	3.725	0.123	0.069
	S3 4 × 4_1.5	1.904	0.433	0.654	3.622	0.517	0.076
B2	S1 4 × 4_1.0	3.559	0.422	0.526	3.140	0.302	0.040
	S2 4 × 4_1.0	4.037	0.401	0.467	3.195	0.086	0.037
	S3 4 × 4_1.0	3.147	0.463	0.583	3.085	0.477	0.043
C1	S1 3 × 3_1.5	2.867	0.437	0.574	3.210	0.511	0.050
	S2 3 × 3_1.5	3.154	0.434	0.527	3.256	0.122	0.047
	S3 3 × 3_1.5	2.678	0.462	0.607	3.177	0.553	0.052
C2	S1 3 × 3_1.0	4.722	0.451	0.527	2.951	0.323	0.027
	S2 3 × 3_1.0	5.245	0.425	0.482	2.993	0.091	0.025
	S3 3 × 3_1.0	4.546	0.462	0.543	2.936	0.476	0.028

**Table 7:** Results of regression analysis ^1^.

	*G_th_*	*G_cont_*	*m_th_*	*ϕ_th_*	*χ_th_*	*m_cont_*	*ϕ_cont_*	*χ_cont_*	*C_str_*	*σ_th_*	*σ_cont_*	*σ_str_*
*G_th_*	1.000	**0.459**	0.160	0.318	0.134	−0.342	−0.318	−0.243	−0.350	**−0.473**	**−0.717**	**−0.435**
*G_cont_*	**0.459**	1.000	−0.374	−0.141	**0.716**	0.027	0.141	0.284	0.141	0.105	**−0.684**	0.110
*m_th_*	0.160	−0.374	1.000	**0.903**	**−0.738**	**−0.804**	**−0.903**	**−0.933**	**−0.953** ^(3)^	−0.043	0.352	−0.020
*m_cont_*	−0.342	0.027	**−0.804**	**−0.979**	**0.602**	1.000	**0.979**	**0.938**	**0.924** ^(3)^	0.054	−0.074	0.030
*χ_th_*	0.134	**0.716**	**−0.738** ^(2)^	**−0.688** ^(2)^	1.000	**0.602**	**0.688**	**0.797**	**0.646** ^(3)^	0.025	**−0.568**	0.014
*χ_cont_*	−0.243	0.284	**−0.933**	**−0.985**	**0.797**	**0.938** ^(2)^	**0.985** ^(2)^	1.000	**0.967** ^(3)^	0.048	−0.267	0.024
*ϕ_th_*	0.318	−0.141	**0.903**	1.000	**−0.688**	**−0.979**	**−1.000**	**−0.985**	**−0.978** ^(3)^	−0.055	0.162	−0.029
*ϕ_cont_*	−0.318	0.141	**−0.903**	**−1.000**	**0.688**	**0.979**	1.000	**0.985**	**0.978** ^(3)^	0.055	−0.162	0.029
*C_str_*	−0.350	0.141	**−0.953**	**−0.978**	**0.646**	**0.924**	**0.978**	**0.967**	1.000	0.055	−0.163	0.027
*σ_th_*	**−0.473**	0.105	−0.043	−0.055	0.025	0.054	0.055	0.048	0.055	1.000	**0.623**	**0.998**
*σ_cont_*	**−0.717**	**−0.684**	0.352	0.162	**−0.568**	−0.074	−0.162	−0.267	−0.163	**0.623**	1.000	**0.619**
*σ_str_*	**−0.435**	0.110	−0.020	−0.029	0.014	0.030	0.029	0.024	0.027	**0.998** ^(1)^	**0.619** ^(1)^	1.000

^1^ Significant statistical correlation coefficients are in bold.

**Table 8:** Results of the statistical analysis for three groups: S1, S2, and S3 ^1^.

Parameter	K–W Test		Post Hoc Test	*p*-Value
*G_th_*	Value of test statistic *p*-value	7.3801 **0.0250**	S1–S2 ^2^ S1–S3 ^2^ S2–S3 ^2^	1.0000 **0.0520** **0.0602**
*G_cont_*	Value of test statistic *p*-value	12.7836 **0.0017**	S1–S2 ^2^ S1–S3 ^2^ S2–S3 ^2^	**0.0602** 0.7026 **0.0013**

^1^ Significant difference in groups is in bold. ^2^ Compared pairs of groups.

**Table 9:** Results of the statistical analysis for three groups: 5 × 5, 4 × 4, and 3 × 3 ^1^.

Parameter	K–W Test		Post Hoc Test	*p*-Value
*m_th_*	Value of test statistic *p*-value	6.2222 **0.0446**	3 × 3–4 × 4 ^2^ 3 × 3–5 × 5 ^2^ 4 × 4–5 × 5 ^2^	0.8384 **0.0386** 0.4792
*m_cont_*	Value of test statistic *p*-value	8.8538 **0.0120**	3 × 3–4 × 4 ^2^ 3 × 3–5 × 5 ^2^ 4 × 4–5 × 5 ^2^	0.4792 **0.0088** 0.3505
*χ_th_*	Value of test statistic *p*-value	1.0643 0.5873	3 × 3–4 × 4 ^2^ 3 × 3–5 × 5 ^2^ 4 × 4–5 × 5 ^2^	– – –
*χ_cont_*	Value of test statistic *p*-value	7.9064 **0.0192**	3 × 3–4 × 4 ^2^ 3 × 3–5 × 5 ^2^ 4 × 4–5 × 5 ^2^	0.4792 **0.0148** 0.4792

^1^ Significant difference in groups is in bold. ^2^ Compared pairs of groups.

**Table 10:** The phases’ exponents for designed woven structures.

Phase Exponent		*m_th_* (-)	*m_th_* (-)	*m_cont_* (-)	*m_cont_* (-)
Width of strip		1.0 cm	1.5 cm	1.0 cm	1.5 cm
	3 × 3	4.84 (8%)	2.90 (8%)	2.96 (1%)	3.21 (1%)
Structure type	4 × 4	3.58 (12%)	2.06 (9%)	3.14 (2%)	3.67 (1%)
	5 × 5	2.79 (14%)	1.52 (19%)	3.38 (2%)	4.50 (3%)

## Data Availability

Experimental methods and results are available from the author.

## References

[1] Tyurin I.N., Getmantseva V.V., Andreeva E.G. (2019). Van der Pauw method for measuring the electrical conductivity of smart textiles. Fibre Chem..

[2] Castano L.M., Flatau A.B. (2014). Smart fabric sensors and e-textile technologies: A review. Smart Mater. Struct..

[3] Gonçalves C., da Silva A.F., Gomes J., Simoes R. (2018). Wearable e-textile technologies: A review on sensors, actuators and control elements. Inventions.

[4] Korzeniewska E., Krawczyk A., Mróz J., Wyszyńska E., Zawiślak R. (2020). Applications of smart textiles in post-stroke rehabilitation. Sensors.

[5] Tokarska M., Frydrysiak M., Zięba J. (2013). Electrical properties of flat textile material as inhomogeneous and anisotropic structure. J. Mater. Sci. Mater. Electron..

[6] Neruda M., Vojtech L. (2012). Verification of surface conductance model of textile material. J. Appl. Res. Technol..

[7] Liu S., Tong J., Yang C., Li L. (2017). Smart e-textile: Resistance properties of conductive knitted fabric—Single pique. Text. Res. J..

[8] Gunnarsson E., Karlsteen M., Berglin L., Stray J. (2015). A novel technique for direct measurements of contact resistance between interlaced conductive yarns in a plain weave. Text. Res. J..

[9] Kazani I., De Mey G., Hertleer C., Banaszczyk J., Schwarz A., Guxho G., Van Langenhove L. (2011). Van Der Pauw method for measuring resistivities of anisotropic layers printed on textile substrates. Text. Res. J..

[10] Tokarska M. (2016). New concept in assessing compactness of woven structure in terms of its resistivity. J. Mater. Sci. Mater. Electron..

[11] Jiyong H., Xiaofeng Z., Guohao L., Xudong Y., Xin D. (2016). Electrical properties of PPy-coated conductive fabrics for human joint motion monitoring. Autex Res. J..

[12] Tokarska M., Gniotek K. (2015). Anisotropy of the electroconductive properties of flat textiles. J. Text. Inst..

[13] Vasile S., Deruck F., Hertleer C., De Raeve A., Ellegiers T., De Mey G. (2017). Study of the contact resistance of interlaced stainless steel yarns embedded in hybrid woven fabrics. Autex Res. J..

[14] Tokarska M. (2017). Mathematical model for predicting the resistivity of an electroconductive woven structure. J. Electron. Mater..

[15] Hertleer C., Meul J., De Mey G., Vasile S., Odhiambo S.A., Van Langenhove L. (2020). Mathematical model predicting the heat and power dissipated in an electroconductive contact in a hybrid woven fabric. Autex Res. J..

[16] McLachlan D.S. (1986). Equations for the conductivity of macroscopic mixtures. J. Phys. C Solid State Phys..

[17] McLachlan D. (1987). An equation for the conductivity of binary mixtures with anisotropic grain structures. J. Phys. C Solid State Phys..

[18] Archie G.E. (1942). Electrical resistivity log as an aid in determining some reservoir characteristics. Trans. AIME.

[19] Glover P.W.J. (2010). A generalized Archie’s law for n phases. Geophysics.

[20] Glover P.W.J., Walker E. (2009). Grain-size to effective pore-size transformation derived from an electrokinetic theory. Geophysics.

[21] Glover P.W.J., Hole M.J., Pous J. (2000). A modified Archie’s law for two conducting phases. Earth Planet. Sci. Lett..

[22] Verwer K., Eberli G.P., Weger R.J. (2011). Effect of pore structure on electrical resistivity in carbonates. AAGP Bull..

[23] Kennedy W.D., Herrick D.C. (2012). Conductivity models for Archie rocks. Geophysics.

[24] Cai J., Wei W., Hu X., Wood D.A. (2017). Electrical conductivity models in saturated porous media: A review. Earth Sci. Rev..

[25] Tokarska M. (2020). Modeling of electro-conductive properties of woven structure based on mixing model. Commun. Dev. Assem. Text. Prod..

[26] Glover P.W.J. (2017). A new theoretical interpretation of Archie’s saturation exponent. Solid Earth.

[27] Berrezueta E., González-Menéndez L., Ordóñez-Casado B., Olaya P. (2015). Pore network quantification of sandstones under experimental CO_2_ injection using image analysis. Comput. Geosci..

[28] ISO/IEC Guide 98-3:2008(En). Uncertainty of Measurement—Part 3: Guide to the Expression of Uncertainty in Measurement (GUM:1995). https://www.iso.org/obp/ui/#iso:std:iso-iec:guide:98:-3:ed-1:v2:en.

[29] Tokarska M. (2019). Characterization of electro-conductive textile materials by its biaxial anisotropy coefficient and resistivity. J. Mater. Sci. Mater. Electron..

[30] Kostajnšek K., Urbas R., Dimitrovski K. (2019). A new simplified model for predicting the UV-protective properties of monofilament pet fabrics. Autex Res. J..

[31] Textiles and Textile Products—Electrically Conductive Textiles—Determination of the Linear Electrical Resistance of Conductive Tracks.

[32] Meul J. (2015). Study of Electro-Conductive Contacts in Hybrid Woven Fabrics. Master’s Thesis.

[33] Test Method for Electrical Surface Resistivity of Fabrics.

[34] ISO 139:2005. Textiles—Standard Atmospheres for Conditioning and Testing. https://www.iso.org/obp/ui/#iso:std:iso:139:ed-2:v1:en.

[35] Corder G.W., Foreman D.I. (2009). Nonparametric Statistics for Non-Statisticians: A Step-By-Step Approach.

